# RNA sequencing identifies common pathways between cigarette smoke exposure and replicative senescence in human airway epithelia

**DOI:** 10.1186/s12864-018-5409-z

**Published:** 2019-01-09

**Authors:** Hannah Voic, Xiuying Li, Jun-Ho Jang, Chunbin Zou, Prithu Sundd, Jonathan Alder, Mauricio Rojas, Divay Chandra, Scott Randell, Rama K. Mallampalli, Yohannes Tesfaigzi, Tyrone Ryba, Toru Nyunoya

**Affiliations:** 1 Division of Natural Sciences, New College of Florida, Sarasota, FL USA; 2 Department of Medicine, University of Pittsburgh, NW628 UPMC Montefiore, 3459 Fifth Avenue, Pittsburgh, PA 15213 USA; 3 VA Pittsburgh Healthcare System, Pittsburgh, PA USA; 4 Cardiovascular Institute, Department of Medicine, Allegheny Health Network, Pittsburgh, PA USA; 5 Vascular Medicine Institute, Department of Medicine, University of Pittsburgh, Pittsburgh, PA USA; 6 Department of Cell and Molecular Physiology, University of North Carolina, Chapel Hill, NC USA; 7 Lovelace Respiratory Research Institute, COPD program, Albuquerque, NM USA

**Keywords:** Replicative senescence, Primary human bronchial epithelial cells, RNA-seq, Cigarette smoke

## Abstract

**Background:**

Aging is affected by genetic and environmental factors, and cigarette smoking is strongly associated with accumulation of senescent cells. In this study, we wanted to identify genes that may potentially be beneficial for cell survival in response to cigarette smoke and thereby may contribute to development of cellular senescence.

**Results:**

Primary human bronchial epithelial cells from five healthy donors were cultured, treated with or without 1.5% cigarette smoke extract (CSE) for 24 h or were passaged into replicative senescence. Transcriptome changes were monitored using RNA-seq in CSE and non-CSE exposed cells and those passaged into replicative senescence. We found that, among 1534 genes differentially regulated during senescence and 599 after CSE exposure, 243 were altered in both conditions, representing strong enrichment. Pathways and gene sets overrepresented in both conditions belonged to cellular processes that regulate reactive oxygen species, proteasome degradation, and NF-κB signaling.

**Conclusions:**

Our results offer insights into gene expression responses during cellular aging and cigarette smoke exposure, and identify potential molecular pathways that are altered by cigarette smoke and may also promote airway epithelial cell senescence.

**Electronic supplementary material:**

The online version of this article (10.1186/s12864-018-5409-z) contains supplementary material, which is available to authorized users.

## Background

Aging is a complex process associated with progressive decline in multiple organ functions [1]. The aging process can be altered by some lifestyle factors, such as smoking. Cigarette smoking accelerates aging-associated shortening of telomeres [2, 3] and increases risk for age-associated diseases, including chronic obstructive pulmonary disease (COPD) [4].

Increase in the number of senescent cells, which are metabolically active but unable to divide, may play a causative role in the development of tissue and organ dysfunction and age-associated diseases through several mechanisms, including an altered secretory phenotype and lack of cell proliferation [5, 6].

Fibroblasts have been extensively used in in vitro models of cellular senescence to determine various endpoints, such as population doublings, telomere length [7], and changes in the transcriptome [8]; however, the effects of cellular senescence on primary human bronchial epithelial cells (pHBECs) have been less studied, likely due to lesser availability, greater expense, and limited population doublings. In tissue culture, normal human lung fibroblasts and pHBECs irreversibly lose proliferative capacity after roughly 50 and 10 population doublings, respectively [9, 10]. This process, referred to as replicative senescence, appears to be caused by attrition of telomeres, as telomerase activation increases the length of telomeres and life-span in normal human cells [11]. Genotoxic stresses such as γ-irradiation can also induce a cellular senescence known as stress-induced premature senescence [12].

Cigarette smoke (CS) exposure is also sufficient to induce cellular senescence both in vitro and in vivo. CS extract (CSE) activates the two canonical senescence-inducing pathways including the p53 and p16-retinoblastoma protein pathways in cultured normal human lung fibroblasts [13]. Furthermore, senescent alveolar type 2 epithelial cells are increased in smokers with COPD relative to smokers without COPD [14], suggesting a potential role of cellular senescence in the pathogenesis of COPD.

The antagonistic pleiotropy concept postulates that some genes are beneficial early in life at the cost of aging [5]. In this study, we hypothesize that some genes beneficial for cell survival in response to CS contribute to the development of cellular senescence. To identify candidate genes and pathways connecting replicative senescence to CS exposure, we used RNA-seq to examine relationships between expression responses in each condition.

## Results

### Identification of expression changes upon replicative senescence or CSE exposure in pHBECs

**Fig. 1:**
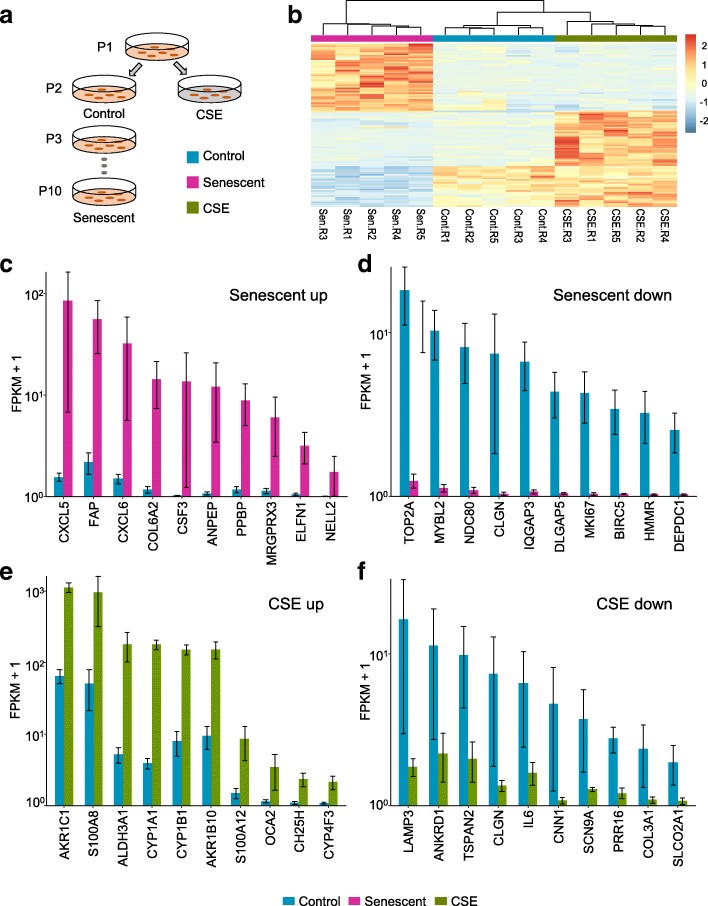
Identification of expression changes upon replicative senescence or CSE exposure in pHBECs. **a** Experimental design. pHBECs were exposed to 1.5% CSE for 24 h at second passage, or passaged into replicative senescence. The clip art of petri dishes was depicted by our own work. **b** Hierarchical clustering of genes (rows) with transcriptional changes in control, senescent, and CSE conditions (columns). Values show standard deviations from average expression values per gene. **c** Top 10 genes upregulated from pHBECs in senescence. Bars show normalized and log-scaled mean expression scores (FPKM+ 1) ± SEM across biological replicates (*n* = 5), with genes ordered by average expression difference between senescence and control expression levels. **d** Top 10 genes downregulated in pHBECs in senescence. **e** Top 10 genes upregulated in pHBECs in response to CSE. **f** Top 10 genes downregulated in pHBECs in response to CSE

To investigate potential shared mechanisms connecting the effects of CSE and cellular senescence, we examined transcripts by RNA-seq from pHBECs cultured from five nonsmokers, as they were passaged into replicative senescence or when treated with CSE at an early passage (Fig. 1a; Additional file 1: Table S1). Transcripts were measured after 24 h treatment in 1.5% CSE (*n* = 5), in equivalent unexposed cells (control; *n* = 5), and in cells that had stopped cell proliferation for 2 weeks after nine to eleven passages (Senescence; *n* = 5). Although the original experimental design included a combined condition to study interaction effects, toward replicative senescence, we did not have enough cells to evaluate the effects of CSE on senescent cells (data not shown). After transcript levels were quantified and normalized, hierarchical clustering of genes with stable differences in expression (Fig. 1b) showed separated experimental replicates by condition, demonstrating that treatment-specific differences in the transcriptome could be readily discovered.

To validate the senescent phenotype, we first examined transcript levels of two known senescence-regulating genes, CDKN2A (p16) and LMNB1 (lamin B1) [15, 16] in late-passage pHBECs. CDKN2A (p16) and lamin B1 showed a 6.5-fold induction and 26-fold repression respectively, consistent with their roles in cellular senescence (Additional file 1: Table S2).

In total, 1534 genes were differentially expressed upon replicative senescence beyond an FDR of 0.05, including 877 transcripts with reduced and 663 with increased expression, representing a robust transcriptional response.

To confirm the reliability of our methods for measuring expression changes through RNA-seq, we selected panels of genes with significant (FDR < 0.05) expression differences in senescent or CSE conditions and compared the consistency of their changes in expression to those measured using quantitative real-time PCR (Additional file 2: Figure S1). Overall, RNA-seq and qPCR methods showed a quite consistent direction and extent of fold-changes in each condition. These included up- and downregulated genes across a wide dynamic range, and RNA-seq showed only a slight tendency to underestimate changes measured by PCR. Therefore, our original quantification by RNA-seq was able to reliably identify relative expression differences between conditions.

Genes with the highest measurable fold increases in senescence are shown in Fig. 1c. These included > 60-fold increases for two CXC-motif chemokines (CXCL5 and CXCL6), for which overexpression is associated with aging in human prostate fibroblasts [17]. These CXC genes, along with granulocyte colony stimulating factor 3 (CSF3) (mean fold increase > 400 in senescence, but high variation) have also been linked to increased expression in COPD [18] and genetic variations in lung function decline in smokers [19]. In addition, FAP, fibroblast activation protein, associated with fibroblast senescence [20], was increased 46-fold in HBEC senescence. ELFN1, a gene linked to disrupted cell cycle control and silenced by Myc-regulated long noncoding RNAs [21] was increased 38-fold in senescence samples. Overall, 177 genes showed > 4-fold expression increases upon replicative senescence (Additional file 1: Table S2).

Genes downregulated in late-passage cells (Fig. 1d) were likewise connected to known mechanisms of senescence. TOP2A encodes a DNA topoisomerase that protects telomeres in cooperation with TRF2, a shelterin member [22] that was reduced 65-fold in senescent cells. Other genes included cell cycle progression and cellular aging marker [23, 24] MYBL2 (B-Myb; 72-fold down), kinetochore complex component NDC80 (73-fold down), putative oncogenic protein DLGAP5 (70-fold down), and MKI67, a widely used proliferation marker [25] (81-fold down). DEPDC1, previously observed to be 12.4-fold downregulated upon loss of p300 in a process that induces cellular senescence in melanoma cells [26], here was approximately 60-fold downregulated. More genes were downregulated upon replicative senescence than upregulated, with 312 and 776 genes reduced in transcript levels more than 4-fold and 2-fold, respectively.

To test cellular responses to CS, cells from the same starting cultures were exposed to 1.5% CSE for 24 h (Fig. 1a), and transcripts were collected by RNA-seq. After CSE exposure, 599 genes were differentially expressed at FDR < 0.05, including 189 transcripts with increased and 412 with reduced expression levels (Additional file 1: Table S1). Consistent with previous studies of the human airway transcriptome [27, 28], genes sharply induced upon CSE exposure (Fig. 1e) included AKR1C1, AKR1B10, and ALDH3A1, enzymes which catalyze aldehyde reduction and oxidation, respectively [29, 30], as well as several subunits of cytochrome P450 (CYP1A1, CYP1B1, CYP4F3), which has broad activity in cellular responses to free radical carcinogens in CS [31, 32].

As in replicative senescence, reductions in gene expression upon CSE exposure appeared greater than inductions (Fig. 1f). These included LAMP3 (22-fold down in CSE), linked to a poor prognosis for malignancies [33]; ANKRD1 (9-fold down), a transcriptional cofactor required for wound repair [34]; TSPAN2 (9-fold down), a transmembrane protein that regulates cell motility [35]; and CLGN (19-fold down), a testis-specific endoplasmic reticulum chaperone protein [36]. IL-6 has several immune and inflammatory functions connected to NF-κB activity and tumorigenesis [37–39], and was 9.1-fold reduced upon CSE exposure, consistent with other CS exposure models [40, 41]. In contrast to the effects of CS on increased gene expression, there was no significant expression change among these downregulated genes in smokers’ airway epithelial cells [27, 28].

### Substantial overlap between replicative senescence- and CSE-induced gene expression changes

**Fig. 2:**
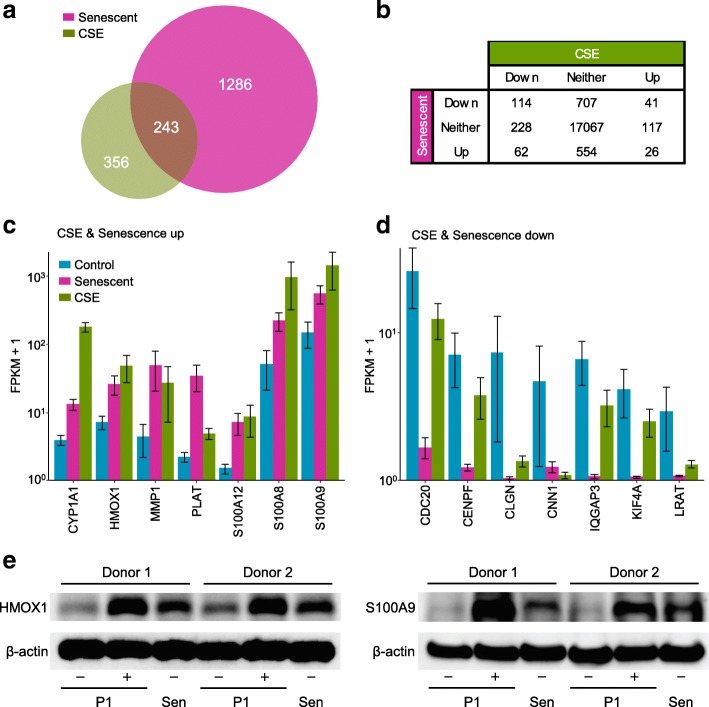
Significant overlap between replicative senescence- and CSE-induced gene expression changes. **a** Overlap in genes differentially regulated versus control in CSE-treated and senescent pPHBECs. **b** Number of genes significantly up- or downregulated at FDR < 0.05 in CSE or senescence conditions. **c** Genes upregulated in both CSE and senescence conditions. Bars show normalized and log-scaled mean expression scores (FPKM+ 1) ± SEM. **d** Genes downregulated in both CSE and senescence conditions. **e** Immunoblot analysis for HMOX1 and S100A9 in control, CSE-exposed (both P1: passage 1) or senescent (Sen) pHBECs isolated from two donors

To study connected pathways and relationships between replicative senescence and responses to CS, we assessed the degree of shared responses in each condition. We first compared the proportion of genes differentially expressed in both senescence and CSE to the proportion of overlap expected in switching genes (8.2% of CSE, 3.2% of senescence) if there were no relationship between conditions. Overall, genes changing expression state upon CSE exposure showed close correspondence with those regulated upon senescence (Fig. 2a). Among the 599 differentially expressed transcripts in CSE treatment, 243 (41%) overlapped with those differentially expressed genes in senescence, representing a 5-fold enrichment above the 49 overlapping genes expected in two random lists (*p* < 1e^− 10^ by Fisher exact test; Additional file 1: Table S3).

We next examined the directionality of this relationship (Additional file 2: Figure S2) to determine whether genes upregulated in one condition were more often upregulated in the other, or vice-versa. Shared downregulation was twice as common as shared upregulation (28% vs. 14% of CSE affected genes, respectively) but this is likely because downregulated genes were more common in both conditions (Fig. 2b). Genes regulated in one condition were differentially expressed in roughly equal proportions in the other, consistent with no relationship in directionality (Additional file 2; Figure S1b; *p* = 0.88 by Fisher test).

Genes with similar responses in CSE and senescence may be driven by common elements in upstream signaling pathways, and those most upregulated or downregulated in both conditions (replicative senescence and CSE) are shown in Fig. 2c and d, respectively. Genes with shared upregulation included CYP1A1, a main cytochrome P450 enzyme involved in procarcinogen activation [42], which was 4.4-fold up in senescence and 57.4-fold in CSE. The central oxidative stress response gene HMOX1 was 4.3-fold elevated in senescence, 7-fold after CSE. Both MMP1, involved in extracellular matrix regulation and tissue remodeling, and PLAT, tissue plasminogen activator, belong to SASP [43]. S100A12, S100A8, S100A9, encoding calgranulin C, A, B, respectively, are involved in neutrophil chemotaxis and adhesion. To validate protein expression in comparison to the RNA seq data, pHBECs from another two healthy donors (43 year-old and 53 year-old males) were cultured, treated with or without 1.5% CSE for 24 h or were passaged into replicative senescence. Steady-state protein levels of HMOX1 and S100S9, two robustly upregulated genes (Fig. 2c) were increased in both CSE-exposed and senescent cells (Fig. 2e). These data suggest that both CSE and replicative senescence can be linked to increased protein levels from the transcriptional activity.

Among genes downregulated in both conditions, both CDC20 and CENPF regulate chromosome segregation and mitosis [44, 45]. CLGN and CNN1, in the top 10 downregulated genes by senescence, were also downregulated by CSE. IQGAP3 is required for cell proliferation [46] and was 77.6-fold down in senescence, 2.6-fold in CSE. KIF4A, involved in microtubule regulation [47], was 52.2-fold down in senescence and 2.2-fold down in CSE. LRAT, involved in vitamin A metabolism, was 24.7-fold down in senescence and 7.1-fold down in CSE.

Overall, 114 genes with attenuated expression upon replicative senescence also showed significantly reduced transcript levels after CSE. Among these, forkhead box protein FOXO1 is associated with signaling through interleukin proteins and CEBPA, and here was reduced 3.8-fold in senescence and 2.4-fold down in CSE (FDR < 0.05).

### Shared pathway enrichments in CSE and senescence responsive genes

**Fig. 3:**
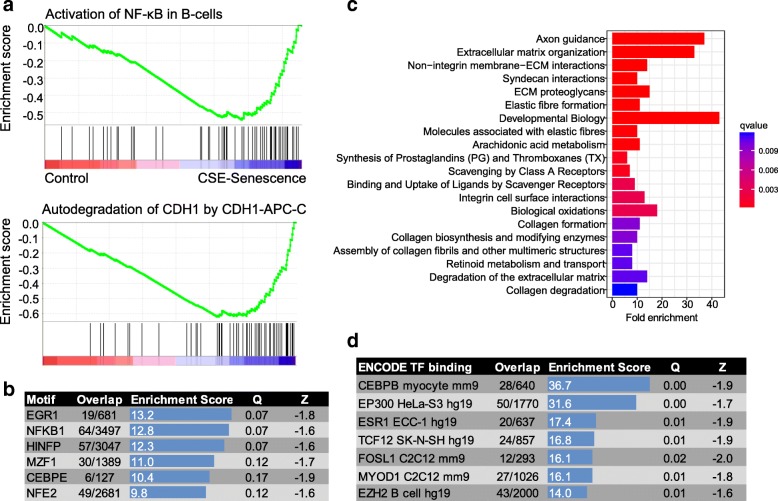
Shared pathway enrichments in CSE and senescence responsive genes. **a** Running enrichment (green) via GSEA for genes associated with NF-κB activation in B-cells (top), and autodegradation of CDH1 by APC-C (bottom). Black lines indicate positions of genes in each pathway along an axis of expression from control values (left, red) to CSE and senescence values (right; blue). **b** TRANSFAC and JASPAR binding motif enrichments in CSE and senescence regulated gene promoters. **c** Enrichment in Reactome biological pathways for CS/senescence regulated genes. **d**. ENCODE transcription factor binding site enrichments near associated CS/senescence genes in mouse (mm9) and human (hg19) tissues and cell lines

Next, to more comprehensively identify upstream molecular mechanisms for genes and pathways regulated in replicative senescence and CSE, we applied a gene set enrichment analysis (GSEA) to CSE and senescence gene lists (Fig. 3a, Additional file 2: Figure S3). Of 4202 gene sets tested, 222 were found associated with CSE-exposed or senescent conditions at a nominal FDR < 0.25, and 93 at FDR < 0.1. The top gene sets (FDR < 0.01; Table 1) were connected to responses to ROS, the ubiquitin-proteasome and phagosome pathways, autodegradation of CDH1, and NF-κB activation, among others. We note that p- and FDR values for pathway enrichment statistics are here measured competitively relative to other pathways rather than other conditions, as distinguished by Goeman and Buhlmann [48]. Enrichments in pathways and annotations can be driven by a smaller core networks of genes, and correlation structures in gene networks inflate nominal FDR estimates of methods that assume independent features. However, GSEA does retain relatively high power and accurate FDR estimates in these situations [49].

**Table 1:** Top GSEA enriched gene sets

Gene set	Source	ES	NES	Q	Lead edge
Reactive oxygen species	Houstis et al.	0.770	2.34	0.0008	63%
Proteasome pathway	BIOCARTA	0.758	2.21	0.0012	91%
Proteasome	KEGG	0.679	2.2	0.0011	90%
ER Phagosome pathway	REACTOME	0.637	2.15	0.0010	81%
Autodegradation of CDH1 by CDH1 APC-C	REACTOME	0.641	2.15	0.0008	76%
Activation of NF-κB in B cells	REACTOME	0.631	2.14	0.0007	67%
Vif-mediated degredation of APOBEC3G	REACTOME	0.656	2.11	0.0011	72%
Cross presentation of soluble exogenous antigens	REACTOME	0.65	2.11	0.0010	70%
MYC targets	Menssen et al.	0.631	2.11	0.0010	66%
Destabilization of mRNA BY AUF1 hnRNP D0	REACTOME	0.639	2.10	0.0012	73%
Antigen processing cross presentation	REACTOME	0.594	2.09	0.0013	69%
p53 independent G1-S DNA damage checkpoint	REACTOME	0.631	2.08	0.0015	68%
CDK mediated phosphorylation and removal of CDC6	REACTOME	0.638	2.07	0.0016	71%
Regulation of ornithine decarboxylase ODC	REACTOME	0.628	2.05	0.0023	73%
SCF-β-TRCP mediated degradation of EMI1	REACTOME	0.615	2.04	0.0025	72%
MYC up	Bild et al.	0.514	2.04	0.0025	67%
Autodegradation of the E3 ubiquitin ligase COP1	REACTOME	0.634	2.04	0.0024	70%
Rapamycin response down	Peng et al.	0.488	2.02	0.0029	68%
NFE2L2 targets	Singh et al.	0.793	2.00	0.0041	90%

FDR-adjusted significance (Q), enrichment scores (ES), normalized enrichment scores (NES) are shown for MSigDB gene sets with FDR < 0.01 enrichment. Lead edge signal represents the proportion of matching genes in the early, continually rising portion of the enrichment score

We tested enrichment among additional categories of biological features and annotations for the 243 genes with shared differential expression in both conditions by applying Fisher exact tests through Enrichr [50]. Enriched gene ontologies and biological pathways were consistent with numerous smoking and senescent phenotypes, including pathways involved in the regulation of cellular division and replication (Additional file 2: Figure S4). Genes containing binding motifs for transcription factors EGR1, MZF1, NF-κB, and HINFP were enriched beyond FDR < 0.1 (Fig. 3b; Additional file 3: Table S5). Several pathways in the Reactome database [51] were highly enriched in terms related to senescence and CS responses, including fibrosis, oxidative stress, and connections to the extracellular matrix (Fig. 3c). For transcription factor protein binding in ENCODE cell lines and tissues, shared CS/senescence response genes were frequently bound by TF proteins CEBPB, EP300, and EZH2 (Fig. 3d; Additional file 3: Table S6).

### Distinct components of the CSE and replicative senescence expression response

**Fig. 4:**
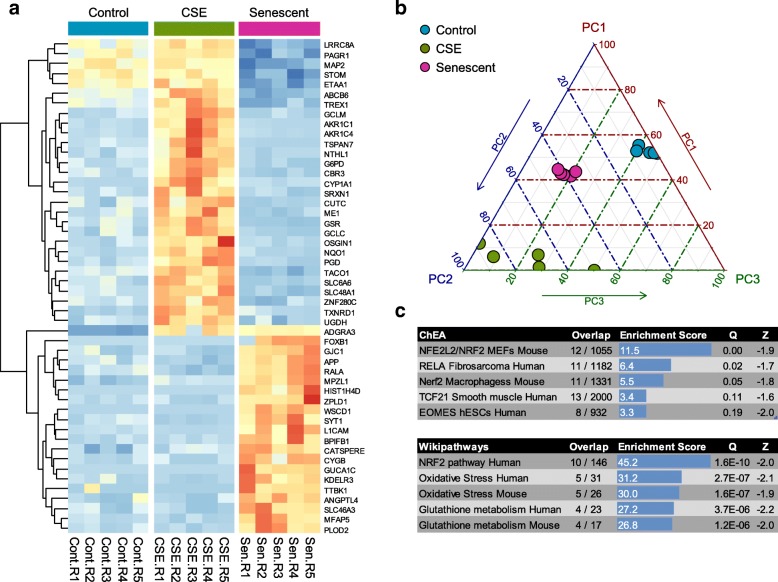
Distinct components of the CSE and replicative senescence expression response. **a** Hierarchical clustering of genes with the top 50 most stable expression changes between control, CSE-exposed, and senescent condition. Relative expression levels per gene are shown, from low (blue) to high (red). **b** Principal component analysis of clustering in conditions using genes in a). Ternary plot shows positions along three PC axes in each condition. **c** Enrichments among chIP-seq genomic targets of transcription factors (ChEA; upper panel) and biological pathways (Wikipathways; lower panel) in stably switching genes

While many senescence-associated genes were induced by CSE, portions of the replicative senescence and CSE response remained distinct. To examine this, we narrowed the lists of differentially expressed genes to those with especially stable expression changes between conditions. These genes readily separated samples in CSE-exposed and senescent conditions from controls by hierarchical clustering (Fig. 4a) and principal component analysis (PCA; Fig. 4b). Such genes may distinguish mechanisms of cellular stress response in replicative senescence from those in oxidative stress, or identify genes normally involved in detoxification and triggered upon CS exposure, but lost once cells senesce.

We then tested for pathway and feature enrichments among genes with the top 50 most stable differences in transcript levels (Fig. 4c, Additional file 1: Table S4). Among these, 12 may be explained through induction by Nrf2/NFE2L2, a master regulator of cellular redox status. Other notable genes in the top 200 most stable changes included thioredoxin reductase 1 (TXNRD1; 1.6-fold increase in senescent cells, 5.6-fold increase by CSE) and glutathione disulfide reductase (GSR; 2.3-fold decrease in senescent cells, 2.9-fold increase by CSE), which are both involved in the detoxification of ROS [52, 53].

To evaluate the robustness of enrichments and directionality of expression changes, we applied self-contained gene set tests on these pathways using ROAST [54]. These demonstrated that the pathways identified through competitive gene set tests were also strongly associated with differential gene expression in self-contained tests that account for inter-gene correlation structure, with 8 of 8 and 14 of 18 FDR < 0.2 gene sets significant after multiple testing correction for those with numerous differentially expressed genes (Additional file 3: Tables S5-S8), and 14 of 31 for those with fewer genes (Additional file 3: Tables S9, S10).

**Fig. 5:**
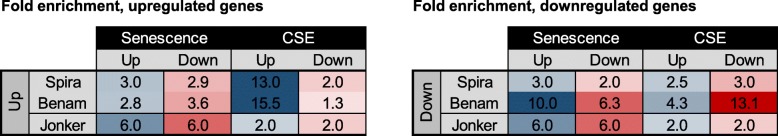
Fold-enrichment among overlaps in gene sets identified in the present study compared to previous studies of CS [27, 52] and senescence [53]

Finally, we characterized how expression changes in our experimental system compare to those of previous studies of CSE [27, 55] and senescence [56] to determine how our results relate to those in other experimental systems (Fig. 5). Of genes differentially regulated upon CS treatment by Spira et al. [27], 39 were found to overlap the equivalent set in our experiment. This reflects a nominal 13-fold enrichment above the 3 overlapping genes expected from unassociated lists of the same size. Likewise, we saw a 15.5-fold enrichment against the equivalent upregulated genes from Benam et al. [55], with a closer connection to the latter for downregulated genes. For senescence, 6 of 10 genes with FDR < 0.05 increases or decreases in expression were conserved between our results and a previous study [56], representing at least 6-fold enrichment. Overall, these results suggest that a sizable fraction of top-ranking genes are robustly identified by separate experimental conditions and groups.

## Discussion

While several aspects of environmental exposures in cellular aging have been well studied, a deeper understanding of molecular pathways that connect senescence to CS exposure remains elusive. It is clinically evident that continuous smoking accelerates aging and shortens lifespan associated with a high prevalence of COPD, pulmonary fibrosis, and lung cancer [57–59]. Understanding these connections remains an important goal from both basic biological and public health perspectives.

In this study, we applied RNA-seq to identify shared pathways and regulators that may explain portions of the cell aging response to CSE exposure in pHBECs. While portions of the transcriptional response in primary bronchial cells may be distinct from cells in vivo, this approach identified genes affected by CSE that are well-known regulators of senescence response and COPD pathogenesis, as well as novel potential therapeutic targets and upstream regulators. For example, CYP1A1, HMOX1 and MMP1, among the top 7 upregulated genes in both senescence and CSE, have been recognized as encoding senescence-associated proteins upregulated by CS [43, 60–62]. Some genetic variations of these three genes are also associated with COPD [63–65]. CYP1A1, xenobiotic monoxygenase, is required to attenuate hyperoxia-induced lung inflammation. HMOX1 is cytoprotective against CS [66] and required to protect against cadmium-induced emphysema [67]. CYP1A1 and HMOX1 are transcriptionally regulated by the aryl hydrocarbon receptor (AHR) and nuclear factor-erythroid 2 like 2 (NRF2) in response to xenobiotics and oxidative stress, respectively [68]. AHR and NRF2 have been also implicated as proteins playing a protective role in smoking-induced COPD [69, 70]. However, most of other transcriptional targets of AHR and NRF2 are upregulated by CSE, but not by senescence in our study. The differential gene expression is likely due to the involvement of other transcriptional factors and/or epigenetic mechanisms. In contrast to a protective role of the three upregulated genes, collagenase MMP1 may play a causative role in the development of COPD, because MMP1 overexpression is sufficient to cause pulmonary emphysema in vivo [71]. The MMP1 overexpression commonly seen in various types of senescence may be attributable to AP-1 and NF-κB, which are each known to drive its expression [72, 73]. Likewise, the upregulation of calgranulin family proteins (S100A8, S100A9, S100A12) may be detrimental by aggravating airway inflammation in COPD through the receptor for advanced glycation end products (RAGE) [74]. Although most robustly upregulated genes in both senescence and CSE function in a stress response, most robustly downregulated genes appear to be involved in the regulation of cell cycle and/or microtubule/cytoskeleton. These downregulated genes have not been reported to associated with COPD to our knowledge.

Although our primary focus is to identify shared gene alteration by replicative senescence and CSE, we also observe differentially regulated genes between the two conditions (62 genes up by senescence and down by CSE and 41 genes down by senescence and up by CSE). These gene sets may also be useful to uncover important targets for cell senescence. For example, GCLC was 3.9-fold up by CSE, but 1.8-fold down in senescence. GCLC, a known transcriptional target of Nrf2, is the catalytic subunit of glutamate cysteine ligase required to synthesize glutathione. Senescence-associated downregulation of these stress response genes may contribute to the development of age-associated diseases.

Enrichment analysis in both CSE and senescence related pathways suggested potential regulators that are known to be upstream of many responses in both conditions. For example, NF-κB, MAFK, miR29A, B, C, miR-24, CEBPB, RORA, and EP300 were each enriched in connected pathways in CSE and senescence responding genes and may be promising candidates for promoting cellular aging by CS [75, 76]. Of note, NF-kB, CEBPB, and RORA have been implicated in COPD pathogenesis linked to DNA damage and/or lung inflammation [77–79].

There are several limitations in this study. First, our in vitro senescence model is artificially made by replication and not initially confirmed through biomarkers (e.g., senescence-associated beta galactosidase activity or CDKN2A protein expression). However, we validated this model by known senescence-inducing or associated genes (CDKN2A and LMNB1). In addition, many SASP members are also upregulated in these terminally passaged primary cells. Second, we identified approximately 1500 genes significantly altered by replicative senescence, but most of them are likely changed as the consequence of cell senescence. We will need to further investigate a causative role of the individual changes, especially shared signals between senescence and CSE. In particular, modeling interaction effects with a factorial design may be useful to identify amplified responses in addition to enrichments in shared pathways, once CS-treated cells can be successfully passaged into replicative senescence. In addition, a remaining challenge is to unravel the types of adaptive responses to environmental exposures and the connections between them. Such responses may promote cell survival upon ROS exposure or oncogene activation, or may conversely trigger cell cycle delay, senescence and death [80, 81]. Carefully targeted experiments are needed to further explore potential mechanisms for candidate upstream regulatory genes as the priority.

## Conclusions

Some cellular responses to survive damaging stress from CS may involve a tradeoff to promote cell senescence. In this study, the high overlap in gene responses to replicative senescence and CSE suggests an intimate mechanistic relationship between CS exposure and cellular aging. Our results identify promising links among pathways regulating ROS, proteasome degradation, and NF-κB signaling that may contribute to smoking-induced cellular aging.

## Methods

### Cell culture

pHBECs were acquired from Universities of North Carolina and Pittsburgh. pHBECs were isolated from five individuals aged 12–65 and from smokers and non-smokers, three of whom were males, without known lung diseases; these lungs were not suitable for transplantation. Further, validation of protein expression was performed on pHBECs that were isolated from another two male nonsmoking donors aged 43 and 52 without known lung diseases. Bronchial epithelial cells were characterized, isolated, and cultured as previously described [82]. The population doubling of each pHBECs (from passage 1 [P1]) was monitored in p100 dishes (100 mm) at a starting cell density of 15 × 10^3^/cm^2^. Once pHBECs achieve 80–90% confluency, the cells were split into two p100 dishes until no additional cell growth was observed for 2 weeks.

### Preparation of cigarette smoke extract (CSE)

100-mm research cigarettes (3R4F) were purchased from the University of Kentucky. CSE solutions were prepared as previously described [83]. pHBECs at P2 or pHBEC2 cells were exposed to 1.5% CSE for 24 h after plating.

### RNA-Seq

Total RNA was extracted from pHBEC cultures using trizol as previously described [84]. Briefly, oligo-dT magnetic beads were bound to the crude RNA fraction of cell homogenate. RNA was then eluted from the beads using a mix of zinc-fragmentation buffer with elution buffer and random primers. The random primer cDNA was synthesized and fragments were adenylated at the 3′ position. Illumina DNA multiplex barcoded oligonucleotide adapters were ligated to the cDNA in a sample specific manner to allow multiple samples to be pooled [85]. The cDNA sequencing libraries were amplified by 15 cycles of PCR. Quality of the cDNA was assessed with 2100 Bioanalyzer microfluids using the RNA 600 Nano kit (Agilent Technologies), and cDNA libraries were stored at − 20 C. An Illumina HiSeq 2000 Genome analyzer was then used for multiplex sequencing of the samples at the Biofrontiers Institute of Advanced Genomics (University of Colorado, Boulder, CO). A total of 30–35 million 2 × 50 reads per sample were generated. The raw sequences were aligned to the NCBI human nuclear genome (build 37) using TopHat [86]. The read quality of the samples was checked and normalized using the RNA-SeQC method [87].

### RNA-Seq analysis

RNA-seq datasets were processed using the Tuxedo suite of RNA analysis tools [86], as implemented through the Galaxy Project (http://www.usegalaxy.org). Cufflinks was used to determine individual transcript abundance in each sample using an algorithm [86]. Samples were analyzed individually in this software, and the expression values were assembled and normalized against each other using Cuffcompare. Samples were then tested for differential gene and transcript expression using Cuffdiff, the output data of which were visualized in the cummeRbund R package [86].

### Real-time PCR quantification

Threshold cycles were converted to relative expression levels using the ddCT method. Fold changes between conditions were averaged across two internal controls (ACTB and GAPDH) and five biological replicates per condition.

### Gene set enrichment and pathway analysis

Using the expression data output from Cuffdiff, GSEA was used to determine what, if any, gene sets were enriched in expression differences between conditions. Leading edge subset analysis (a determination of genes shared between multiple significant gene sets) was performed on the GSEA output to identify potential candidate genes which may help examine whether cigarette exposure and senescent phenotypes are similarly regulated in any way. Significant genes identified by cummeRbund were tested for enrichment in pathway and gene ontology databases using Enrichr [50], using the Z-score of the match as a measure of significant set enrichment. Of the genes found to be differentially expressed similarly between both senescent and smoking conditions by the Tuxedo suite, the candidate gene predictor program ToppGene was used to identify which genes may be possible candidate genes for future inquiries. ToppGene is an open source program used to prioritize candidate genes based on their functional annotation. A small training gene set was supplied along with the candidate gene set. Training set genes included genes belonging to the Gene Ontology collection for senescence and genes identified by Spira et al. [27]. The candidate gene set was compared to the training gene set looking for over represented terms in the annotation and the average expression vector for the training gene sets [88].

## Additional files

Additional file 1: RNA-seq values and tests. **Table S1.** Normalized expression values from RNA-seq per transcript. **Table S2.** Genes with significant expression changes upon CSE exposure or senescence. **Table S3.** Genes with significant expression changes (FDR < 0.05) in both CSE and senescence. **Table S4.** Genes with the top 50 most stable expression differences between normal PHBEC, CSE, and senescence conditions. (XLSX 6038 kb)

Additional file 2: **Figure S1.** Validation of RNA-seq expression fold changes. **Figure S2.** Summary of overlaps in CSE and senescence expression changes. **Figure S3.** Enrichment of CS/Senescence expression signatures using GSEA. **Figure S4.** Ontologies and pathways enriched separately upon CS exposure and senescence. (PDF 210 kb)

Additional file 3: Pathway enrichment summary. **Table S5.** JASPAR and TRANSFAC motif enrichments in shared CS and senescence responses. **Table S6.** ENCODE TF binding motif enrichments in shared CS and senescence responses. **Table S7.** JASPAR and TRANSFAC motif enrichment using self-contained methods. **Table S8.** ENCODE TF binding motif enrichments using self-contained methods. **Table S9.** Wikipathways enrichments among top-50 stably expressed genes. **Table S10.** Wikipathways enrichments using self-contained methods. (XLSX 200 kb)

## Abbreviations

AHR: Aryl hydrocarbon receptor
COPD: Chronic obstructive pulmonary disease
CS: Cigarette smoke
CSE: Cigarette smoke extract
FDR: False discovery rate
NRF2: Nuclear factor-erythroid 2 like 2
pHBEC: Primary human bronchial epithelial cell
ROS: Reactive oxygen species
