# Supplementary material for: RNA sequencing identifies common pathways between cigarette smoke exposure and replicative senescence in human airway epithelia
Source: BMC Genomics. 2019 Jan 9;20:22. doi: 10.1186/s12864-018-5409-z (PMC6325884; doi:10.1186/s12864-018-5409-z)
Supplement: Supplementary file 2 — Figure S1. Validation of RNA-seq expression fold changes. Figure S2. Summary of overlaps in CSE and senescence expression changes. Figure S3. Enrichment of CS/Senescence expression signatures using GSEA. Figure S4. Ontologies and pathways enriched separately upon CS exposure and senescence. (PDF 210 kb) [file 12864_2018_5409_MOESM2_ESM.pdf]

Figure S1

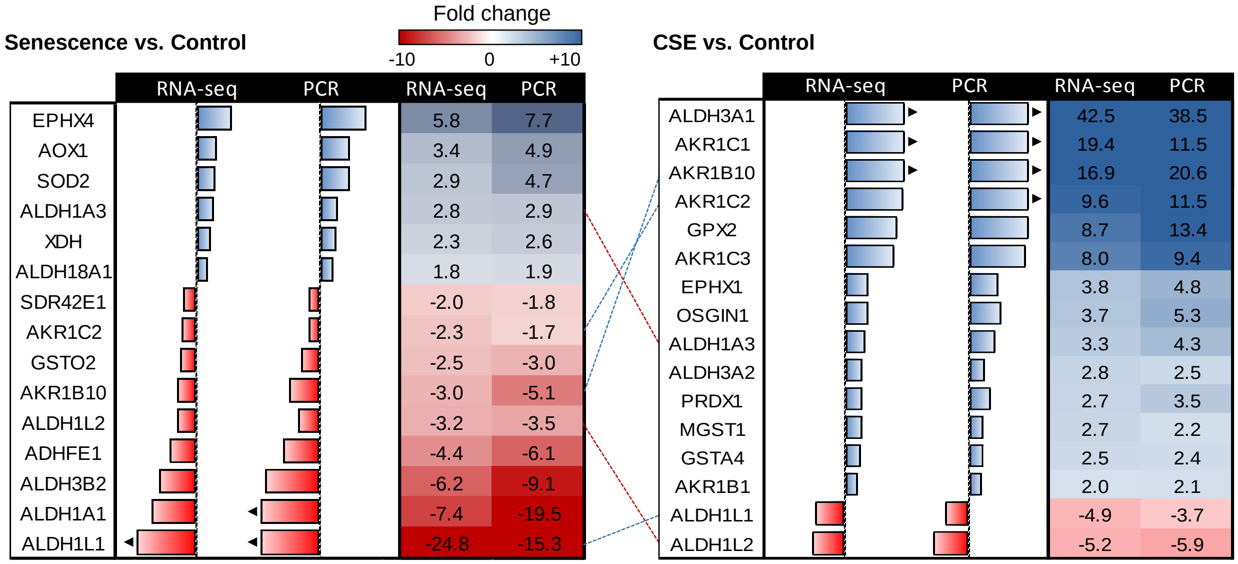

Figure S1. Validation of RNA-seq expression fold changes.

Comparison of expression fold-changes by RNA-seq and quantitative real-time PCR for genes with significant (FDR < 0.05) changes in expression upon replicative senescence (left) or CSE exposure (right). Bars reflect relative fold changes from control to experimental conditions, up to ±10-fold (with greater changes indicated by '►'). Dashed lines connect genes with significant expression changes in both conditions.

Figure S2

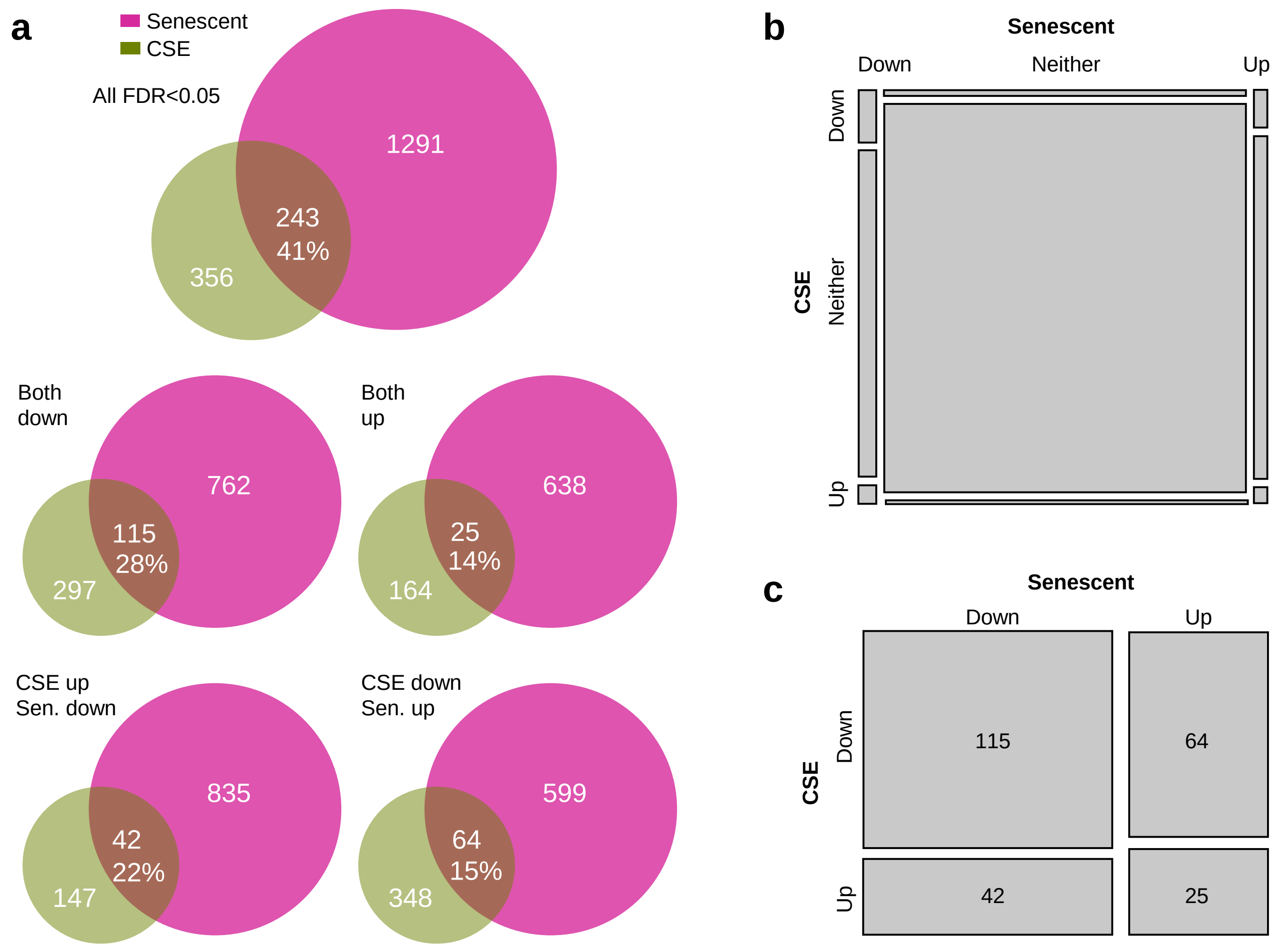

Figure S2. Summary of overlaps in CSE and senescence expression changes.

a) Overlap in genes differentially regulated versus control in CSE-treated and senescent PHBECs, highlighting the percentage of replicative senescence-regulated genes also differentially expressed upon CSE exposure.

b) Mosaic plot shows the relative number of genes in each regulation state in CSE and senescence conditions relative to control PHBECs.

c) Mosaic as in b, restricted to genes up- or downregulated in both CSE and senescence conditions.

Figure S3

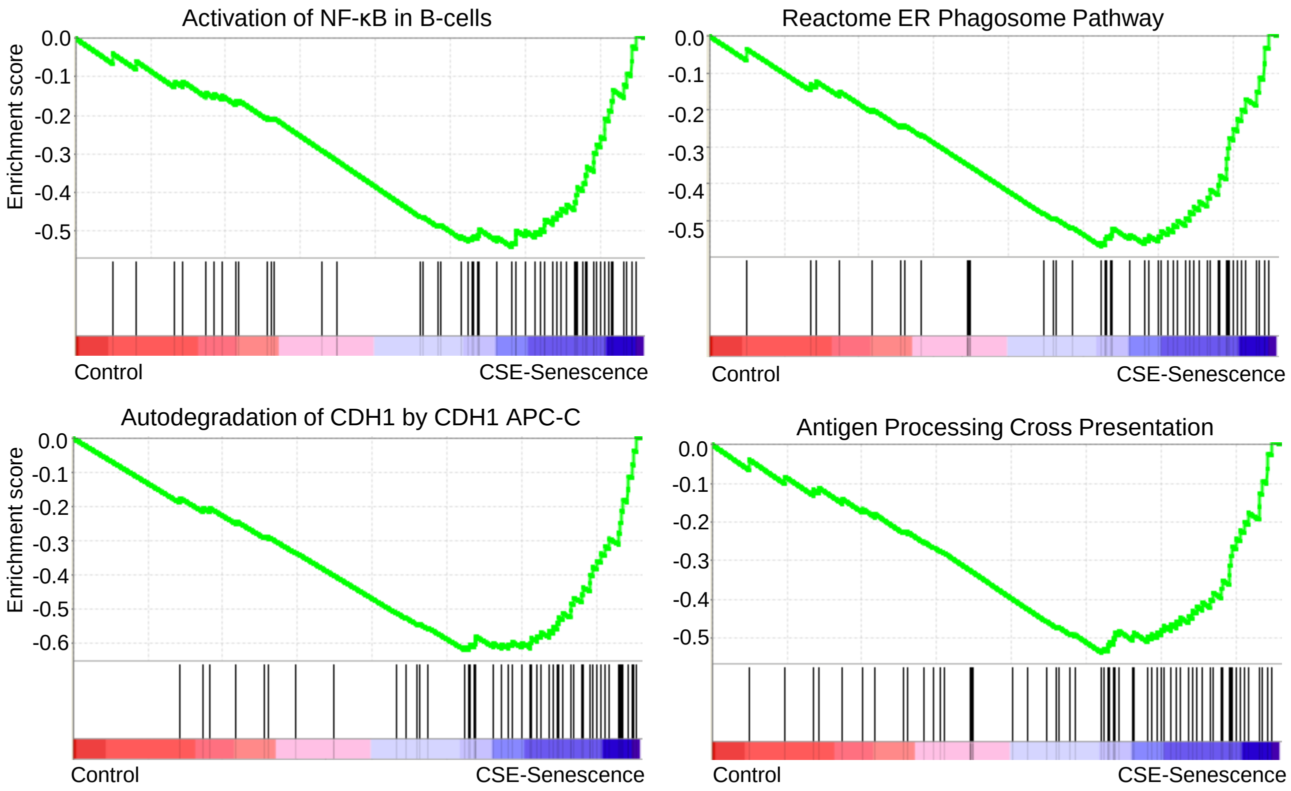

Figure S3. Enrichment of CS/Senescence expression signatures using GSEA.

GSEA running enrichment scores for genes associated with the above labeled pathways are depicted in green lines. Black vertical lines represent genes sorted by signal/noise ratio in CS/Senescence differential expression values.

Figure S4

KEGG

Cigarette smoke		
Term	Overlap	Z
Axon guidance	42/515	-2.34
Developmental Biology	51/786	-2.30
Scavenging by Class A Receptors	7/19	-2.16
Metabolism_Homo sapiens	101/1908	-2.15
Extracellular matrix organization	35/283	-2.11
Metabolism of lipids and lipoproteins	40/659	-2.02
Biological oxidations	21/199	-2.00
Arachidonic acid metabolism	13/53	-2.00
Elastic fibre formation	13/41	-1.96
Collagen formation	12/85	-1.95

Senescence		
Term	Overlap	Z
M Phase	52/266	-2.29
S Phase	33/124	-2.17
DNA strand elongation	14/32	-2.10
G1/S-Specific Transcription	11/17	-2.08
Mitotic Prometaphase	33/108	-2.08
Resolution of Sister Chromatid Cohesion	30/100	-2.05
Mitotic Metaphase and Anaphase	34/173	-2.05
Mitotic G1-G1/S phases	30/135	-2.03
E2F mediated regulation of DNA replication	13/33	-2.01
Extracellular matrix organization	47/266	-2.01

Reactome

Cigarette smoke		
Term	Overlap	Z
Amoebiasis	14/100	-1.82
Arachidonic acid metabolism	10/62	-1.80
Metabolism of xenobiotics by cytochrome P450	12/73	-1.80
Axon guidance	17/127	-1.78
Focal adhesion	19/202	-1.77
Metabolic pathways	66/1239	-1.71
Steroid hormone biosynthesis	10/58	-1.62
Chemical carcinogenesis	12/82	-1.60
PI3K-Akt signaling pathway	23/341	-1.60
TGF-beta signaling pathway	10/84	-1.57

Senescence		
Term	Overlap	Z
bladder cancer	12/40	-1.97
cell cycle	29/104	-1.89
cell communication	23/137	-1.80
axon guidance	21/126	-1.72
pathogenic escherichia coli infection epec	10/48	-1.57
pathogenic escherichia coli infection ehec	10/48	-1.39
glutamate metabolism	7/30	-1.01
natural killer cell mediated cytotoxicity	19/129	-0.95
inositol phosphate metabolism	9/46	-0.93
phosphatidylinositol signaling system	12/72	-0.93

Gene ontology

Cigarette smoke		
Term	Overlap	Z
response to acid chemical (GO:0001101)	26/275	-2.41
response to extracellular stimulus (GO:0009991)	30/313	-2.40
response to nutrient levels (GO:0031667)	28/291	-2.40
cyclooxygenase pathway (GO:0019371)	7/15	-2.38
extracellular matrix organization (GO:0030198)	44/359	-2.38
quinone metabolic process (GO:1901661)	8/29	-2.38
extracellular structure organization (GO:0043062)	44/360	-2.38
response to wounding (GO:0009611)	26/167	-2.37
response to oxidative stress (GO:0006979)	31/290	-2.36
monocarboxylic acid metabolic process (GO:0032787)	43/473	-2.34

Senescence		
Term	Overlap	Z
mitotic sister chromatid segregation (GO:0000070)	12/20	-2.62
sister chromatid segregation (GO:0000819)	13/23	-2.58
response to inorganic substance (GO:0010035)	62/370	-2.46
regulation of mitotic cell cycle (GO:0007346)	69/391	-2.45
regulation of cell adhesion (GO:0030155)	62/336	-2.45
positive regulation of cell cycle (GO:0045787)	48/278	-2.44
regulation of cell cycle process (GO:0010564)	77/481	-2.44
response to oxygen levels (GO:0070482)	44/259	-2.40
response to steroid hormone (GO:0048545)	60/369	-2.40
response to hypoxia (GO:0001666)	42/241	-2.37

Figure S4. Ontologies and pathways enriched separately upon CS exposure and senescence.

KEGG and Reactome pathway enrichments from lists of differentially expressed genes in CSE and senescence are shown, along with gene ontologies and associated Z-scores.
